# Supplementary material for: Development of a computer-aided design software for the quantitative evaluation of aesthetic damage
Source: PLoS One. 2019 Dec 18;14(12):e0226322. doi: 10.1371/journal.pone.0226322 (PMC6919621; doi:10.1371/journal.pone.0226322)
Supplement: S1 Code site — 1. (ZIP) [file pone.0226322.s001.zip › [EESC_jr][Projeto]Relatorio_07_21/vendors/select2/docs/_includes/options/compatibility/matcher.html]

## Simplified function for matching data objects

Added in Select2 4.0.0.
This method was added to make upgrading easier from earlier versions of
Select2.

During the Select2 4.0.0 release, the
`matcher` function was changed to allow for more complex
matching of nested objects.

Key
:   `matcher`

Value
:   A function taking a search `term` and the data object
    `text`.

Adapter
:   `oldMatcher`

The custom matcher example provides a
guide for how to use this in your own application. For those upgrading
from older versions of Select2, you just need to wrap your old
`matcher` with this function to maintain compatibility.
